# When Passive Feels Active - Delusion-Proneness Alters Self-Recognition in the Moving Rubber Hand Illusion

**DOI:** 10.1371/journal.pone.0128549

**Published:** 2015-06-19

**Authors:** Anaïs Louzolo, Andreas Kalckert, Predrag Petrovic

**Affiliations:** 1 Department of clinical neuroscience, Karolinska Institutet, Stockholm, Sweden; 2 Department of neuroscience, Karolinska Institutet, Stockholm, Sweden; Institute of Psychology, GERMANY

## Abstract

Psychotic patients have problems with bodily self-recognition such as the experience of self-produced actions (sense of agency) and the perception of the body as their own (sense of ownership). While it has been shown that such impairments in psychotic patients can be explained by hypersalient processing of external sensory input it has also been suggested that they lack normal efference copy in voluntary action. However, it is not known how problems with motor predictions like efference copy contribute to impaired sense of agency and ownership in psychosis or psychosis-related states. We used a rubber hand illusion based on finger movements and measured sense of agency and ownership to compute a bodily self-recognition score in delusion-proneness (indexed by Peters’ Delusion Inventory - PDI). A group of healthy subjects (n=71) experienced active movements (involving motor predictions) or passive movements (lacking motor predictions). We observed a highly significant correlation between delusion-proneness and self-recognition in the passive conditions, while no such effect was observed in the active conditions. This was seen for both ownership and agency scores. The result suggests that delusion-proneness is associated with hypersalient external input in passive conditions, resulting in an abnormal experience of the illusion. We hypothesize that this effect is not present in the active condition because deficient motor predictions counteract hypersalience in psychosis proneness.

## Introduction

One of the most complex computations the brain has to perform involves construction of the experienced boundary between the body and the external world. This conscious experience of the body in space depends on two basic cognitive processes. The first is the perception of limbs as part of one’s own body—the sense of body ownership—[1–4]. The second is the experience that we are in voluntary control of our bodily actions—the sense of agency [5,6]. Both the sense of ownership and agency contribute to the self-recognition of bodily actions in a variety of contexts and laboratory tasks [7–9].

Psychotic patients have difficulties in precisely identifying the border between the self and the external world—in relation to their experience of both body ownership and agency of their actions [10–14]. Apart from a strange and sometimes bizarre experience of their body and how they interact with the external world, the underlying erroneous processes seem to have a direct relation to positive symptoms such as hallucinations and delusions [15,16].

Based on two different lines of research it has recently been discussed whether schizophrenic patients display a reduced or an increased sense of agency as compared to controls. The comparator model hypothesis suggests that an efference copy is normally used to predict the sensory consequences of an action and to cancel out self-induced perceptions in order to better focus on externally produced input [17,18]. Psychotic patients seem to have a dysfunctional efference copy and cannot reliably distinguish between self- and externally-generated perceptions [19,20]. According to this theory these patients would perceive their movements as originating externally [21,22] and would not feel in control of their actions [23].

Another line of research has shown that schizophrenic patients, when executing a movement, are more likely than controls to attribute the observed movements (based on visual feedback) to themselves, even in cases of large temporal or spatial differences [24,25]—here we refer to this phenomenon as “*over-inclusive agency*”. The comparator model seems not to be compatible with the observations of over-inclusive agency—as it would predict a decreased sense of agency [21,22]. In order to explain this discrepancy recent studies have suggested that hypersalient processing of external sensory input is responsible for the over-inclusive agency effect in schizophrenia patients [15,21,22,26]. In principle this could mean that an altered efference copy might not be related to these effects of over-inclusive agency. However, in the earlier studies this possibility was not directly investigated [21,22]. Thus, the cause of over-inclusive agency remains unclear.

The idea that psychotic patients interpret external sensory inputs differently compared to normal individuals resulting in an abnormal experience of their own body has been investigated using the rubber hand illusion (RHI) [27–30]. In the classical version of this illusion, participants perceive a model hand as part of their body (sense of body ownership) as a result of synchronous strokes applied to the rubber hand in full view and to the participants’ real hand, which is hidden from view [1,3]. Schizophrenic patients report an abnormally vivid experience of ownership and a significantly faster onset of the illusion [27,28]. A similar link between psychosis-related states and enhanced illusory ownership has also been shown in healthy subjects using schizotypy and psychosis-proneness questionnaires [15,16,28,29] and pharmacological manipulations including ketamine [31] or amphetamine [32]. Intriguingly, in psychosis-related states participants report experiencing a stronger illusion in both synchronous and asynchronous visuo-tactile stimulations [28,29], which is perhaps due to over-responsiveness to the visual impression of the rubber hand. These observations are in line with the idea that heightened salience towards external sensory input, visual cues in particular, could explain the generally increased bodily self-recognition in schizophrenic patients and psychosis-related states.

**Fig 1:**
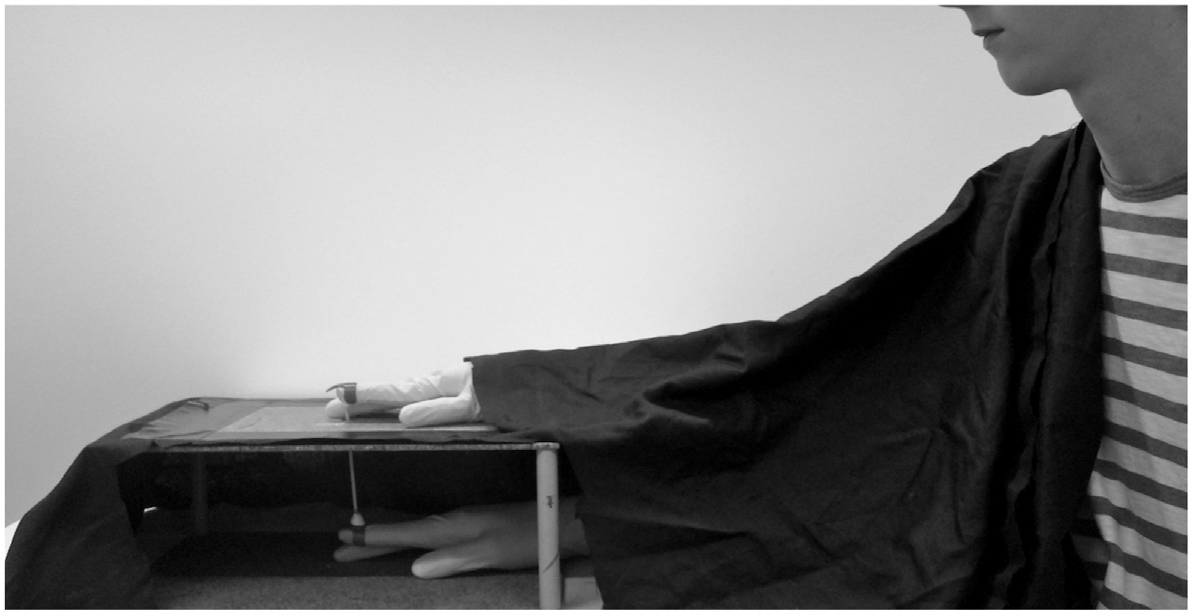
Illustration of the moving rubber hand setup used in the study.

Here we use a recently introduced version of the RHI that is based on finger movements rather than tactile stimulation and therefore involving visual, proprioceptive, and motor cues [33,34]. Crucially, this paradigm allows independent investigation of the sense of body ownership and sense of agency—two key aspects of bodily self-recognition—in a single experimental setup [7,33,35–37]. In this experiment, the index finger of a model hand moves either synchronously or asynchronously with the participants’ hidden real finger through a mechanical coupling device (Fig 1). When participants perceive the model hand’s finger to move in synchrony with their own finger movement, participants experience the illusion of ownership, akin to the classical illusion where the illusion arises a consequence of visuo-tactile synchrony [33,36,37]. The illusion here may stem on perceptual and cognitive mechanisms comparable to the classical rubber hand illusion [34].

The movements of the real and the fake fingers can be actively produced by the participant, thus involve intentions and efference copy mechanisms (active condition), or they can be produced by the experimenter while the participant remains passive, thus excluding these voluntary motor processes (passive condition). In previous studies the active condition was associated with large increases in agency ratings and slight increases in ownership ratings compared with the passive condition [33,34]. This suggests that internal predictions (including efference copy) generated by voluntary motor commands have an impact on agency and possibly ownership experience. Importantly, agency was significantly more modulated than ownership in the active condition, suggesting that these experiences represent different underlying processes [7,33,35,37]

Many core symptoms in clinical psychotic disorders can also be observed to a lesser degree in healthy individuals displaying psychosis- or delusion-proneness [38–41]. In fact, psychosis-proneness is a semi-normally distributed trait in the general population where individuals on the extreme high-end of the spectrum may be characterized by similar cognitive, thought- and perceptual mechanisms as psychotic patients [39–42]. Moreover, individuals exhibiting high psychosis-proneness are also at risk in acquiring a manifest psychotic disorder such as schizophrenia [39,43]. Psychosis-proneness may therefore be used as an experimental model to resolve fundamental questions in psychosis research [44]. At the same time healthy subjects do not suffer from co-morbidity, chronic effects of manifest psychotic disorders on the brain or medication effects—variables that all affect information processing and cognition.

As mentioned above previous studies suggest that hypersalient external input could in principle explain differences in bodily self-recognition (including sense of agency [21,22] and body ownership [27,28]) in psychosis. Although it has also been established that efference copy (or efference copy based comparator mechanisms) is impaired in psychotic conditions [19,20], and in delusion-proneness [42], its contribution to the sense of agency and ownership has not been examined in these states. In the present study we manipulate the involvement of motor predictions, including efference copy, by comparing active and passive movement conditions using the moving rubber hand paradigm as a model for bodily self-recognition [33]. The logic behind this manipulation is that only active movements involve the generation of motor predictions while passive movements do not. Since this experimental manipulation has previously shown strong increases in agency for active vs. passive movements [33,45] we expected that this effect should be smaller in psychosis-related states. As also increased, albeit smaller, ratings have been shown for ownership in active vs. passive movements [33,45] a similar effect could be expected.

## Methods

In order to investigate our hypotheses we tested 71 healthy individuals (30 males; mean age 24.3, range 18–42) using the moving rubber hand illusion paradigm and also examined their delusion-proneness with the Peters’ Delusion Inventory (PDI) [39]. PDI is a self-rating questionnaire that consists of 21 questions focusing on whether the subjects tend to experience any delusion-like symptoms such as paranoid symptoms, grandious symptoms and ideas of reference. The reason for including a relatively large number of subjects was the assumption that there is a low degree of association between subjective ratings of delusion-like experiences and underlying phenoptype. Participants were recruited from the local universities, by open advertisement and were naïve to the purpose of the study. They asserted that they had no neurological or psychiatric disorder. The study (2010/548-31/2 and 2010/1729-32) was approved by the regional ethical review board of Stockholm (www.epn.se). After complete description of the study to the subjects, written informed consent was obtained. Data is accessible under the following repository (https://www.researchgate.net/publication/275957852_PONE-D-14-52234_Results_PDI_and_RHI; DOI: 10.13140/RG.2.1.4394.1286).

### Experimental procedure

Subjects were seated comfortably at a table, where a small wooden box was placed at approx. 50 cm distance. The participant placed his or her hand inside the box with the palmar side on the table. On top of this box a model hand with a movable index finger was placed. Both the participant’s finger and the model hand’s finger were connected with a light stick attached to small finger caps worn on the distal interphalangeal joint. A cloth covered the participant’s right arm to occlude sight from the participant’s real body (see Fig 1) [33].

We tested the group of participants in four conditions where we varied the mode (active, passive movements) and synchronicity of movements (synchronous, asynchronous feedback). Each condition lasted two minutes, where participants made repetitive index finger movements (extension) at approximately 1 Hz. We avoided a regular rhythm as it is known that the illusion works better for varying sequences and therefore sometimes the participants were instructed to make “double taps”, two quick tapping movements in rapid succession. In the passive trials the experimenter moved both the participant’s and the model hand’s fingers by moving the connecting stick. In the asynchronous condition the model hand’s finger moved with a delay of approximately 500 msec. In between each condition participants had a rest period of 45–60 sec, where they stretched and relaxed the right arm. These pauses prevent potential carry-over effects between conditions. All trials were randomized and counterbalanced.

After each trial participants filled in a questionnaire where they rated their experience on a 7 point-Likert scale ranging from “–3” (totally disagree) to “+3” (totally agree), where “0” indicates uncertainty. The questionnaire contains eight statements, with two statements related to the sensation of hand ownership and two statements reflecting the sense of agency of the finger movements. We also included four control statements to control for task compliance (see Supplementary material for details). Statements were adopted from existing questionnaires used in previous rubber hand illusion experiments [3,4,33].

### Hypothesis

In order to test our hypothesis that impaired motor predictions in delusion-prone individuals would impact upon their bodily self-recognition, we measured the subjective ratings in the active conditions (that involved motor predictions) and the corresponding ratings in the passive conditions (that did not involve motor predictions).

In our initial analysis we computed a self-recognition score, in which we averaged both ownership and agency scores. We collapsed synchronous and asynchronous conditions in our primary analysis as both are associated with an efference copy in the active condition but no efference copy in the passive condition, and psychosis is associated with a sense of ownership even when stimulation is asynchronous [28,29]. We hypothesize an increased rating of self-recognition in relation to delusion-proneness in the passive conditions as a consequence of hypersalience to external input. However, we also hypothesized that effect was smaller or non-existent in the active conditions because hypersalience was combined with a weaker influence of motor predictions in relation to delusion-proneness. We further tested how ownership and agency specifically contribute to these differences in bodily self-recognition related to delusion-proneness.

While most studies on delusion-proneness compare groups with high and low delusion-proneness scores we decided to conduct a correlational study over our whole sample. We considered this would give us more reliable information, as criteria for high and low delusion-proneness may differ between studies, which make it difficult to compare results. Another problem using high and low delusion-proneness groups is that differences between the groups may mirror non-linear effects that are not directly associated to the degree of delusion-proneness. Thus, using a correlational approach including the whole sample more accurately reflect the delusion-proneness continuum. Since the data was not normally distributed (Shapiro-Wilk test, p < 0.05) we used non-parametric tests. All results are reported two-tailed. In addition, since we hypothesized delusion-proneness would influence self-recognition differently in the active condition compared to the passive one, we performed a general linear mixed-model analysis to investigate the interaction between PDI scores and the two conditions (the residuals were normally distributed, Shapiro-Wilk test p > 0.05).

## Results

**Fig 2:**
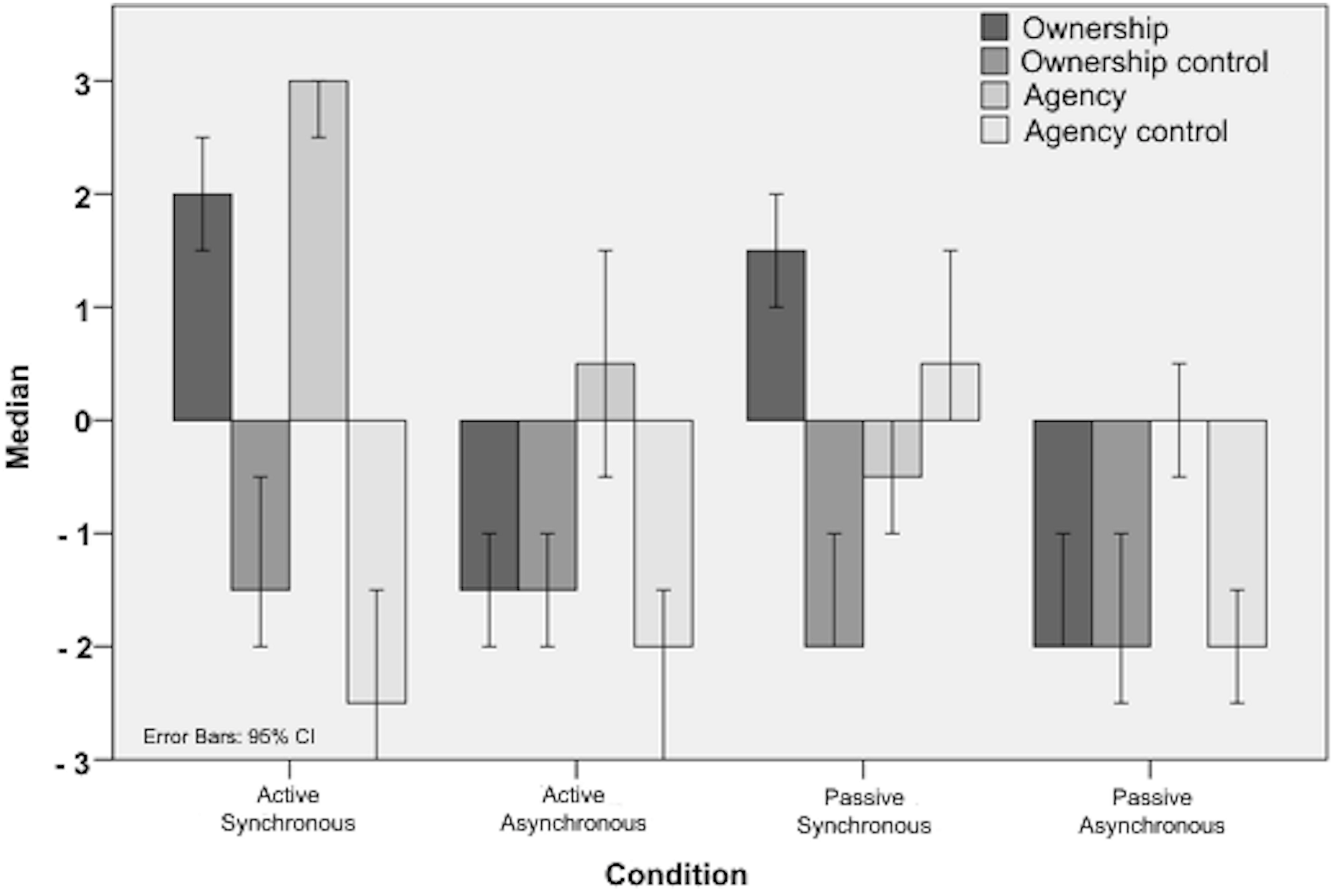
The results of the illusion questionnaire replicate previous results: participants experienced the illusion in both active synchronous and passive synchronous conditions, but not in the asynchronous conditions (Ownership Active Synchronous vs. Ownership Active Asynchronous: Z = -7.036, p < .000; Ownership Passive Synchronous vs. Ownership Passive Asynchronous: Z = -6.804, p < .000). Median and 95% CI is shown. See also S1 File.

First, we examined the questionnaire data to verify successful induction of the moving rubber hand illusion. The basic results (Fig 2 and S1 File) in terms of group rating scores of ownership and agency for each individual condition confirmed successful illusion induction in this group of participants and are in line with previously published results [33,36,45].

**Fig 3:**
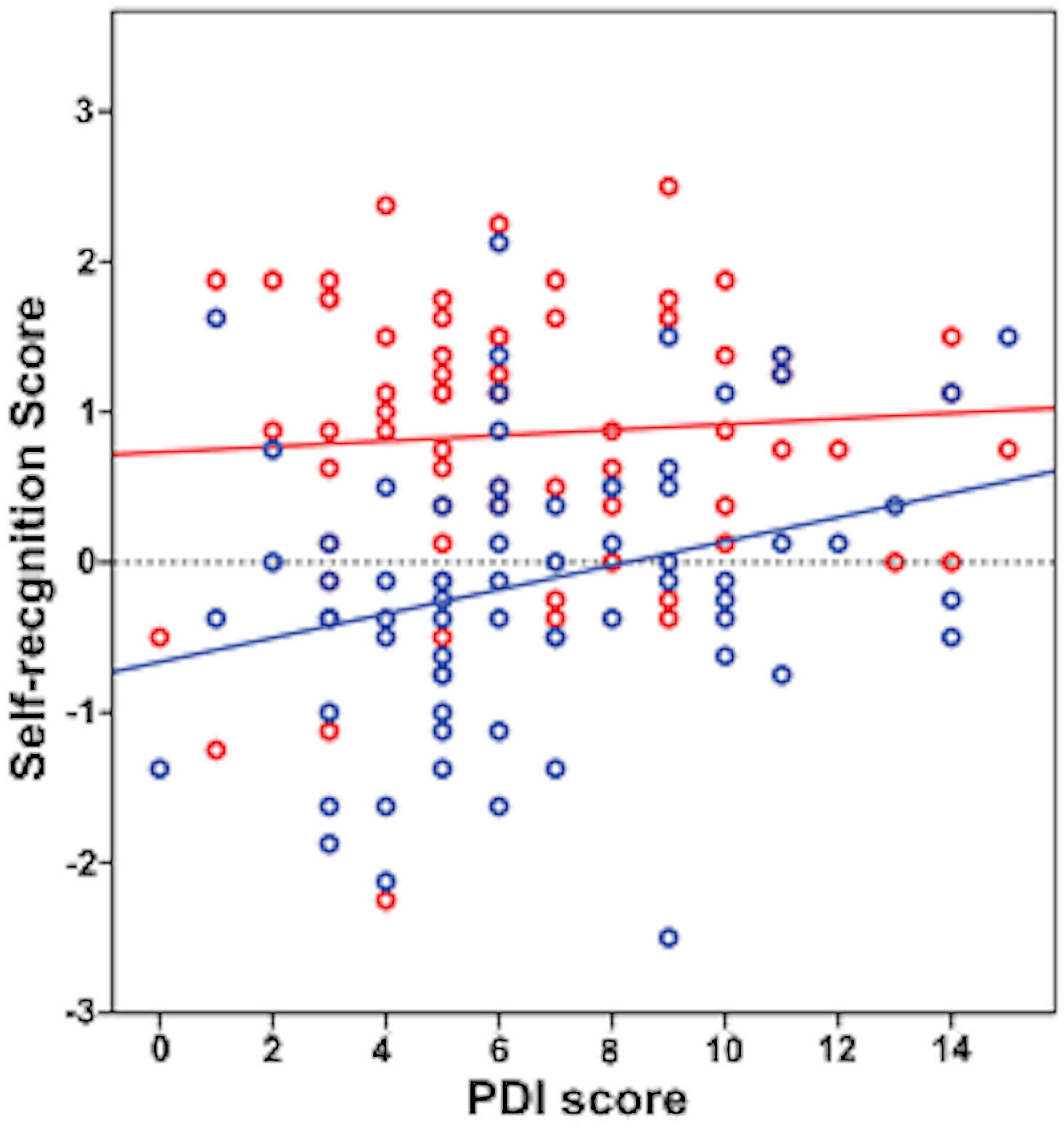
Correlations between the Self-recognition and the PDI score in active (red) and passive (blue) conditions. Spearman’s rank correlation: Active conditions: r = -0.002, p = 0.988. Passive conditions: r = 0.313**, p = 0.008.

To examine our hypothesis of the general relationship between delusion-proneness and body self-recognition we averaged ownership and agency ratings to obtain a single self-recognition score. When we examine the data for active and passive conditions separately we found a significant positive correlation between self-recognition score and PDI in passive conditions (Fig 3, r = 0.313**, p = 0.008; Spearman’s rank correlation), and no significant correlation between self-recognition score and PDI in active conditions (r = -0.002, p = 0.988; Spearman’s rank correlation). This differential effect of PDI on self-recognition between conditions (active/passive) was supported by a general linear mixed model analysis (the conditions were an independent factor and PDI a covariate), showing a tendency towards a significant PDI*conditions interaction (F = 3.102, p = 0.083). This suggests a trend towards a specific effect of PDI on self-recognition in the passive condition.

**Fig 4:**
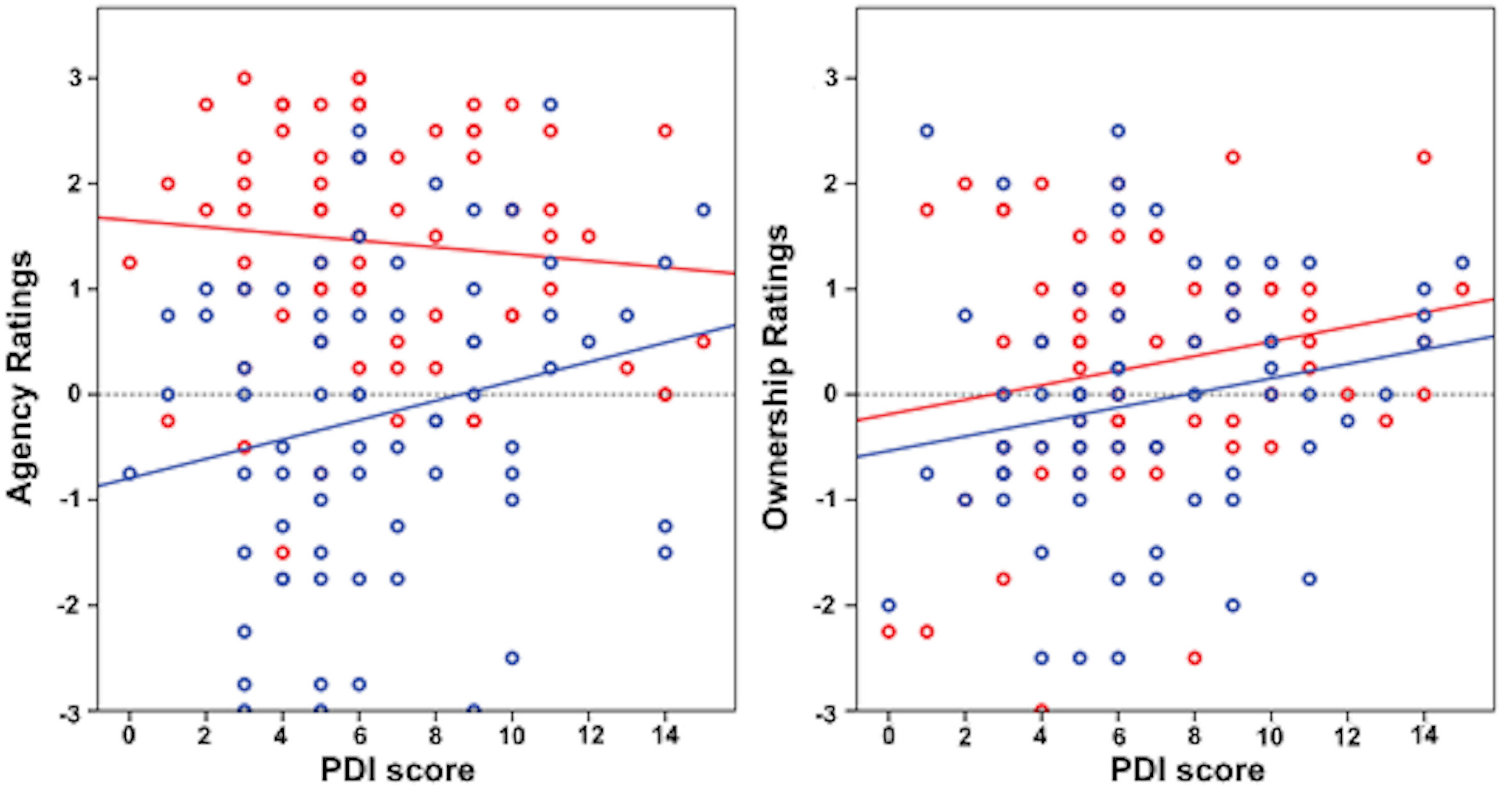
Correlations of the agency (A) and ownership (B) ratings in active (red) and passive (blue) conditions with PDI score: both show a generally abnormal increase with delusion-proneness. A: Active conditions r = -0.111, p = 0.358, Passive conditions r = 0.232*, p = 0.05; B: Active conditions r = 0.177, p = 0.139 Passive conditions r = 0.259*, p = 0.029*.

In order to better understand the data we analyzed ownership and agency ratings separately (see Fig 4). In line with our hypothesis we found that the relationship between agency and delusion-proneness differed between active and passive movements. We observed a positive correlation between agency and PDI scores in passive conditions (r = 0.232*, p = 0.05; Spearman’s rank correlation), but no such relationship in the active movement conditions (r = -0.111, p = 0.358; Spearman’s rank correlation). This specificity of the effect was further confirmed by the linear mixed model analysis, showing a significant interaction between PDI and the active/passive conditions (F = 4.699*, p = 0.034). For ownership we found a significant positive correlation between ownership ratings and PDI scores in the passive conditions (r = 0.259*, p = 0.029; Spearman’s rank correlation) but not in the active conditions (r = 0.177, p = 0.139 Spearman’s rank correlation). However the interaction analysis did not show any significant PDI*conditions interaction (p = 0.995), hence the difference in the PDI/ownership correlation between conditions observed here is not significant.

## Discussion

In the present study we asked how delusion-proneness is associated with the capacity to identify the own body by adding ratings of body ownership and agency into a self-recognition score. We show that delusion-proneness alters self-recognition in the moving rubber hand illusion paradigm. This effect was driven by a robust increase in bodily self-recognition with delusion-proneness in passive conditions, while there was no change in the active conditions (Fig 3). Our observations are in agreement with the idea that motor prediction weakens [14,20,42,46] while external input becomes more salient [21,22] with delusion-proneness.

Our results contribute to the understanding of how failures of bodily self-recognition can arise in the psychosis-prone brain. While it has previously been shown that over-inclusive agency phenomenon in schizophrenia depends on hypersalient external input [21,22] studies have not investigated whether weakened motor predictions, such as efference copy, also alter agency (or ownership). Our results suggest that both components (weakened predictions and hypersalient external input) co-exist and impact upon the sense of agency and ownership in delusion-proneness leading to failures in bodily self-recognition [11].

Remarkably, highly delusion-prone individuals gave equally strong agency ratings in active and passive conditions (see Fig 4), which suggests that participants tend to experience both voluntary active movements and passive movements as self-produced. This implies that these individuals may experience agency in the absence of motor intentions, purely due to heightened reliance on external sensory signals. This increased responsiveness to external input is also reflected in the sense of ownership. Previous studies investigating psychosis or psychosis related states with the rubber hand illusion have shown an abnormally strong ownership experience and faster onset of the illusion [28,29,32]. In sum, this suggests that hypersalient processing of both agency and ownership cues might be related to failures in self-recognition in psychosis related states [11].

It is interesting to consider the possibility that the two mechanisms contributing to the result in the present study (imprecise/weak efference copy and increased salience for external input) are driven by the same underlying malfunctioning neuro-cognitive mechanisms. Indeed, it may be argued that it is unlikely that the factors driving the increase in ownership ratings and in agency ratings, with increasing delusion-proneness (increased salience for external input) constitute two entirely different processes. Rather, they could be related to a more fundamental impairment in the formulation of central predictions across perception-action domains [15,47]. Thus it is possible that the same underlying pathological mechanism may cause changes in both external input and expectations. One such possible fundamental mechanism could be the capacity of higher-order areas to generate central predictions about the state of lower level representations (sensory, motor, or cognitive) as formalized within the predictive coding framework [48]. In this theoretical framework, imprecise or weakened predictions may cause increased salience because of a lack of suppression of the input signals. Alternatively, aberrant or hypersalient input signals may prevent establishment of stable low-level predictions [44]. Stronger faulty signals may build up even more imprecise expectations which would influence belief formation (as proposed for example by a hierarchical Bayesian model of schizophrenia), eventually resulting in delusions [15,47].

Our experiment was performed in healthy individuals and related to psychosis proneness. While psychosis-proneness continuum is an ecologically valid model for a psychosis [49,50] we cannot directly apply the present results to psychotic disorders like schizophrenia. Further investigations with psychotic patients are therefore needed in order to extend these findings to clinical populations. However, there also are several positive implications with studying healthy subjects since this group shows no problems with comorbidity, no effect of drug treatment and no cognitive decline. Another limitation of the study is that our data consist only of questionnaire ratings. Although quantitative psychometric reports of subjective experiences like agency and ownership are clearly relevant to psychiatry, further studies are needed to corroborate our findings with objective measures of ownership and agency. However, it is important to point out that our results cannot be explained by a putative general tendency of delusion-prone participants to score high on questionnaires, because we found specific differences between active and passive conditions and between agency and ownership ratings with respect to delusion-proneness. Similarly, it is less likely that our findings mirror effects of delusion-proneness on general attentional capacity since our data suggests specific interactions between different states and not a general effect on all illusions. In order to avoid non-linear effects not directly relating to the degree of delusions proneness we applied a correlational approach, instead of dividing subjects into high and low delusion-prone subjects. A correlational approach also avoids variable cut-offs that define “high” and “low” delusion-proneness in different studies.

Finally, although active conditions include motor predictions like efference copy, while passive conditions do not, these conditions also differ in terms of other motor-related processes in the generation of voluntary movements (intention, formation of motor commands). A related point is that our data do not allow us to distinguish between effects that are related to efference copy, from effects that are specifically related to the comparison of efference copy signals and sensory feedback. A selective impairment of the latter comparator mechanisms in delusion-proneness would arguably lead to weaker differences between synchronous and asynchronous conditions, but that is not what we found in our data (see S4 File).

In conclusion, the present results contribute to our understanding of the interplay between motor predictions and sensory salience in bodily experiences in delusion-proneness. These observations may help to explain failures of self-recognition in psychotic disorders like schizophrenia.

## Supporting Information

S1 File Analysis of the questionnaire data. (DOCX)

S2 File Descriptive data of the PDI results. Frequency distribution of the PDI (Yes/No) score within our population sample (n = 71) **(Figure A)**. The corresponding descriptive table (Figure B). The PDI (Yes/No) score is not normally distributed in our sample (Shapiro-Wilk test, p = 0.032). This distribution is similar to the distribution in the original study by Peters et al. on the 21-item PDI (39).
(DOCX)

S3 File Correlation in the aynchronous condition. Correlations between the overall self-recognition scores (ownership + agency) in Active (red) and Passive (blue) conditions with PDI score in the asynchronous condition (Figure A). Active condition: r = -0.006, p = 0.960, and Passive condition: r = 0.268**, p = 0.024 (Spearman’s rank correlation).
(DOCX)

S4 File Additional correlations Synchronous versus Asynchronous. (DOCX)
