# Supplementary material for: When Passive Feels Active - Delusion-Proneness Alters Self-Recognition in the Moving Rubber Hand Illusion
Source: PLoS One. 2015 Jun 19;10(6):e0128549. doi: 10.1371/journal.pone.0128549 (PMC4474665; doi:10.1371/journal.pone.0128549)
Supplement: S1 File — (DOCX) [file pone.0128549.s001.docx]

**SI 1: Analysis of the questionnaire data**

**Statements in the RHI questionnaire:**

**Ownership category:**

I felt as if I was looking at my own hand

I felt as if the rubber hand was my hand

**Agency Category:**

The rubber hand moved just like I wanted it to, as if it was obeying my will

Whenever I moved my finger I expected the rubber finger to move in the same way

**Ownership Control Category:**

It seems as if I had more than one right hand

It felt as if I had no longer a right hand, as if my right hand had disappeared

**Agency Control Category:**

I felt as if the rubber hand was controlling my movements

I felt as if the rubber hand was controlling my will

In order to evaluate the participants’ performance and verify the presence of the illusion in our group of participants we performed a general analysis of the questionnaire results independently from the PDI analysis (see also 33, 34).

We tested the presence of an ownership and agency experience, respectively, by comparing each affirmed illusion category (≥1) to the control statement category within one condition (i.e. Ownership category vs. Ownership-control category in the synchronous condition). We also compared the ownership and agency ratings of the synchronous condition to the asynchronous condition, which strongly reduced these experiences in most normal participants and is used as a control condition in rubber hand illusion experiments (i.e. Synchronous Ownership category vs. Asynchronous Ownership category). Wilcoxon signed-rank tests (two-tailed) have been used for these pair wise comparisons. We corrected for multiple comparisons by applying a Bonferroni correction.

**Ownership category:**

Participants rated the ownership category in the active synchronous condition significantly higher than the control category (Ownership vs. Ownership-control: Z=-7.041, p < .000). It was also significantly higher during synchronous than during asynchronous feedback (Ownership Synchronous vs. Ownership Asynchronous: Z=-7.036, p < .000). The same was observed also in the passive condition: the ownership category was significantly higher than the control category (Ownership vs. Ownership-control: Z=-6.85, p < .000) and also significantly higher in the synchronous feedback condition than in the asynchronous feedback condition (Ownership Synchronous vs. Ownership Asynchronous: Z=-6.804, p < .000).

**Agency category:**

We compared the agency category in the same manner. During active synchronous movements participants rated the agency category significantly higher than the control category (Agency vs. Agency-control: Z=-7.246, p < .000) and also higher than during asynchronous feedback (Agency Synchronous vs. Agency Asynchronous: Z=-6.802, p < .000). During the passive condition the agency category was not significantly higher than the control category (Agency vs. Agency-control: Z=-2.134, p = .033) with the significance level set to p = .00625 after Bonferroni correction.

**Additional comparisons: Ownership and agency in active versus passive**

To test if the ratings of the ownership and agency category are indeed higher during active movements than in passive movements we compared the ratings of the active conditions to the passive conditions. We found that the ownership ratings were significantly higher in the active conditions than in the passive conditions (Ownership Active vs. Ownership Passive: Z=-2.370, p = 0.018). Similarly the agency ratings were significantly higher during active movements than in passive movements (Agency Active vs. Agency Passive: Z=-6.869, p < .000).

**Responders:**

Like in previous studies we classified participants as “ownership responders”, when they rated the ownership category equal or higher than 1 (33, 34). Of our sample of 71 participants 61 were classified as responders in the active synchronous condition (85.9%) and 54 in the passive synchronous condition (76%). Similarly, 67 participants were classified as agency – responders (≥1) in the active synchronous (94.4%) and 20 in the passive synchronous condition (28.1%).

Taken together these results show that we were able to reproduce the moving rubber hand illusion in this group of participants (see 33, 34) and these results are in line with other observations on the rubber hand illusion (see 1,3, 33, 34, 37).
