# Supplementary material for: When Passive Feels Active - Delusion-Proneness Alters Self-Recognition in the Moving Rubber Hand Illusion
Source: PLoS One. 2015 Jun 19;10(6):e0128549. doi: 10.1371/journal.pone.0128549 (PMC4474665; doi:10.1371/journal.pone.0128549)
Supplement: S2 File — Frequency distribution of the PDI (Yes/No) score within our population sample (n = 71) (Figure A). The corresponding descriptive table (Figure B). The PDI (Yes/No) score is not normally distributed in our sample (Shapiro-Wilk test, p = 0.032). This distribution is similar to the distribution in the original study by Peters et al. on the 21-item PDI (39). (DOCX) [file pone.0128549.s002.docx]

**SI2: Peter delusional belief questionnaire**

After the rubber hand experiment participants filled in the Peter´s delusional inventory (PDI) (38, 39). We focused on the Yes/No score of the PDI questionnaire as this score evaluates an individuals’ delusion- proneness, while the other PDI ratings (conviction, distress, and preoccupation) focus more on qualitative description of the impact of delusions on participants. In other words, the Yes/No score allows locating the participants on the continuum from normality to psychosis, whereas the other ratings provide information about the characteristics of delusional beliefs. The descriptive data of the PDI distribution among the subjects is given in SI 2Figs. A and B.

**SI 2 Figure A: Descriptive data of the PDI results**

**
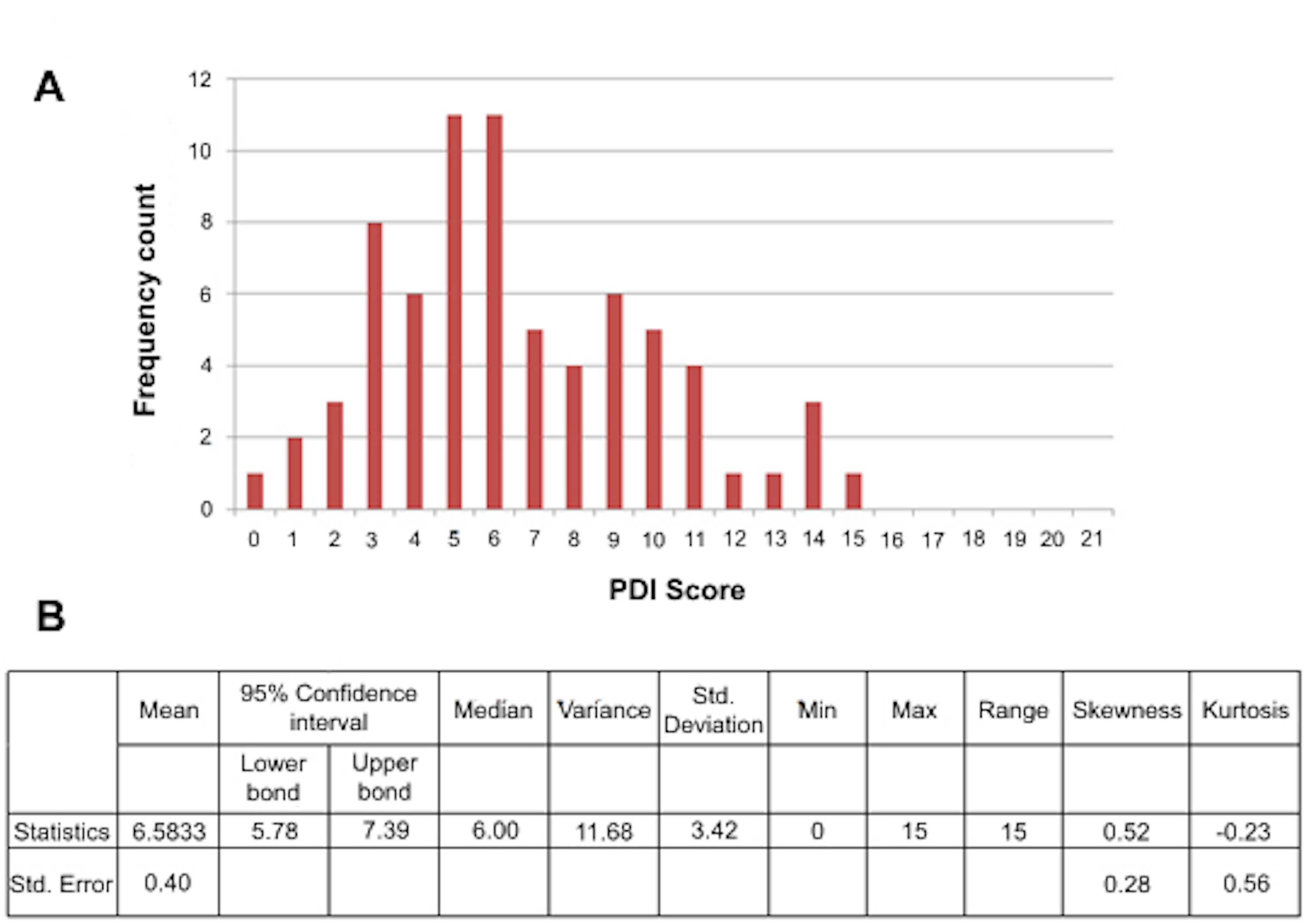
**

Fig. A: Frequency distribution of the PDI (Yes/No) score within our population sample (n=71) Fig. B: the corresponding descriptive table. The PDI (Yes/No) score is not normally distributed in our sample (Shapiro-Wilk test, p=0.032). This distribution is similar to the distribution in the original study by Peters et al on the 21-item PDI (39).
