# Supplementary material for: When Passive Feels Active - Delusion-Proneness Alters Self-Recognition in the Moving Rubber Hand Illusion
Source: PLoS One. 2015 Jun 19;10(6):e0128549. doi: 10.1371/journal.pone.0128549 (PMC4474665; doi:10.1371/journal.pone.0128549)
Supplement: S3 File — Correlations between the overall self-recognition scores (ownership + agency) in Active (red) and Passive (blue) conditions with PDI score in the asynchronous condition (Figure A). Active condition: r = -0.006, p = 0.960, and Passive condition: r = 0.268**, p = 0.024 (Spearman’s rank correlation). (DOCX) [file pone.0128549.s003.docx]

**SI 3: Possible ceiling effect in the correlation between RHI and PDI scores**

A criticism could be that the illusion effect in the active synchronous (but not in the asynchronous) condition is so large for all participants that it yields a ceiling effect, i.e. an illusion score of 3. This could have a confounding impact on the Difference score correlation. Therefore, we also performed a correlation between the Difference score and PDI in just the asynchronous conditions, which in fact closer resembles previous paradigms showing over-inclusive agency in schizophrenia (21,22). This yielded an even more significant effect in line with our hypothesis.

**SI3 Fig. A: Correlation in the asynchronous condition**

A putative ceiling effect in the synchronous conditions was ruled out by repeating the analysis for the asynchronous condition only.

**
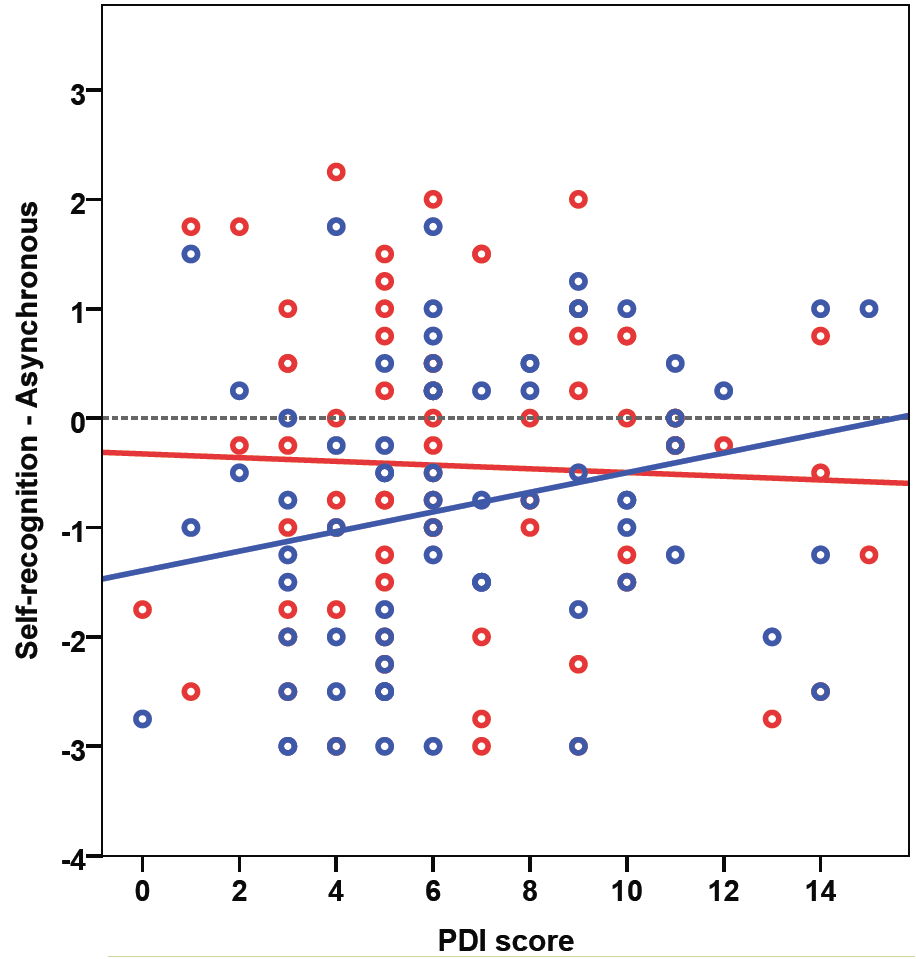
**

**Fig. A.** Correlations between the overall self-recognition scores (ownership + agency) in Active (red) and Passive (blue) conditions with PDI score in the asynchronous condition. Active condition: r = -0.006, p = 0.960, and Passive condition: r = 0.268**, p = 0.024 (Spearman’s rank correlation).
