# Supplementary material for: When Passive Feels Active - Delusion-Proneness Alters Self-Recognition in the Moving Rubber Hand Illusion
Source: PLoS One. 2015 Jun 19;10(6):e0128549. doi: 10.1371/journal.pone.0128549 (PMC4474665; doi:10.1371/journal.pone.0128549)
Supplement: S4 File — (DOCX) [file pone.0128549.s004.docx]

**SI 4: Additional correlations Synchronous versus Asynchronous**

When dividing the data into the synchronous and asynchronous conditions and correlating the resulting ownership and agency ratings to the PDI, respectively we observed no statistically significant relationship, except for the agency score in the synchronous conditions, which showed a trend towards significance (r=0.227 p=0.057): Ownership Synchronous: r=0.157, p=0.191 Ownership Asynchronous: r=0.209,p=0.081; Agency Asynchronous: r=0.057 p=0.635. When we correlated the ownership or agency rating scores from the four individual conditions (active synchronous, active asynchronous, passive synchronous, passive asynchronous) to the PDI the correlations none was significant (p>0.05) except for ownership in the passive asynchronous condition (r=0.254*, p=0.032). These observations are in line with our hypothesis and previous reports of a general increase in self-recognition of the moving rubber hand across the synchronous or asynchronous conditions in highly delusion prone individuals (see introduction), i.e. generally increased ownership ratings in both synchronous and asynchronous condition in psychosis-related states (28-30).
